# Competency and Related Factors in Preventing Emerging Infectious Diseases among Nurses in Long-Term Care Facilities in Taiwan

**DOI:** 10.3390/healthcare10050894

**Published:** 2022-05-12

**Authors:** Po-Jen Kung, Ching-Min Chen

**Affiliations:** 1 Jianan Psychiatric Center, Ministry of Health and Welfare, Tainan City 717, Taiwan; t26084126@gs.ncku.edu.tw; 2 Department of Nursing, College of Medicine, National Cheng Kung University, Tainan City 701, Taiwan; 3 Institute of Gerontology, College of Medicine, National Cheng Kung University, Tainan City 701, Taiwan; 4 School of Nursing, Indiana University, Bloomington, IN 47408, USA

**Keywords:** long-term care facilities, nurses, emerging infectious diseases, pandemic prevention

## Abstract

Emerging infectious diseases (EIDs) are a considerable threat to health, particularly in long-term care facilities (LTCFs), where residents are especially vulnerable. Nurses’ competency in EID prevention is crucial to minimize the adverse effects of EIDs in LTCFs. This study investigated nurses’ competency and related factors in EID prevention in LTCFs in Tainan, Taiwan. A cross-sectional design was employed, and nurses were recruited to complete an online survey examining the knowledge, attitude, and skills required to prevent EIDs in LTCFs. A total of 235 nurses completed the survey. The equivalent score index (SI) for knowledge regarding EID prevention was 68, indicating that the nurses did not have adequate knowledge regarding EID prevention. In contrast, the equivalent SI for the subscale of attitudes toward EID prevention was 78, indicating that the nurses exhibited moderately to highly positive attitudes toward EID prevention. However, they rated themselves as being highly skilled in EID prevention, corresponding to an equivalent SI of 91. Perceived supervisors’ approval, marital status, attitudes toward EID prevention, EID prevention skills, knowledge regarding EIDs, and being in charge of infectious disease prevention were significant predictors of the nurses’ competency. LTCF nurses, especially those working in nursing homes, should enhance their knowledge regarding EID prevention. These findings may help improve nurses’ competency in preventing EIDs by encouraging the integration of practice strategies, education, research, and policy recommendations to eliminate EIDs in LTCFs.

## 1. Introduction

Emerging infectious diseases (EIDs), as defined by the World Health Organization (WHO), are infectious diseases that are newly identified and were previously unknown and cause public health problems either locally or internationally [1]. In 2003, the Institute of Medicine outlined various factors that affect the spread of EIDs; these include microbial adaptation and change, changes in human demographics and behavior, international travel, and the breakdown of public health measures [2]. Increased modes of transportation and speed thereof have enhanced the probability of the sudden outbreak of acute viral respiratory infections. EIDs pose considerable challenges to the global health system and economies [3]. In particular, nurses working at the frontlines may experience substantial challenges.

In 2014, the WHO updated its guidelines for the prevention of epidemic- and pandemic-prone acute respiratory infections (ARIs). Because ARIs are the leading cause of morbidity and mortality from infectious disease worldwide, these guidelines call for preventing EIDs caused by infectious agents such as severe acute respiratory syndrome coronavirus (SARS-CoV) [4]. Coronavirus disease 2019 (COVID-19), the major recent EID, caused by SARS-CoV-2 is an acute respiratory infection. The outbreak of COVID-19 was declared a pandemic by the WHO in March 2020, and as of 2022, confirmed COVID-19 cases and deaths are still high or increasing in many parts of the world. Because of insufficient knowledge regarding the disease at the beginning of the outbreak, most countries failed to introduce effective measures to curb the pandemic, and many frontline medical professionals were infected [5]. In October 2021, the International Council of Nurses (ICN) announced that more than 1.8 million medical professionals worldwide were infected with COVID-19 and that 6643 nurses had died from COVID-19 [6]. The ICN estimated that on average, nearly 10% of the confirmed cases in the world were medical workers, indicating that medical workers and their patients had a considerably high risk of infection than the general public [7].

Healthcare workers and residents of long-term care facilities have a higher risk of dying from COVID-19. Cluster infections in a medical institution can even result in the rapid service breakdown of surrounding medical facilities and thus affect the entire local medical system [8]. With the ongoing spread of COVID-19 worldwide, long-term care facilities in Taiwan are no exception. Confirmed COVID-19 infections and even deaths among the staff and residents of these facilities have been frequently reported [9].

In Taiwan, the 156th local COVID-19 case was the first long-term care facility nurse to be infected with COVID-19. Although the infection occurred during the peak of the pandemic worldwide, the nurse disregarded her symptoms, did not seek immediate medical attention, and continued to work until she received a diagnosis of COVID-19. This nurse did not have sufficient knowledge regarding the necessary measures to prevent infectious diseases and was reluctant to take precautions. In contrast, government agencies were more active in managing the case. The experience of this COVID-19 case indicated that once the professionals or residents of a long-term care facility are infected with an EID, it is likely to develop into a situation where the infection of one person can shut down the entire facility, thus multiplying the social costs of managing the outbreak [10].

Studies have suggested that the residents of long-term care facilities have an increased risk of infection because of their age, declining physical functions, and underlying diseases [11]. Moreover, because long-term care facility nurses work on the frontline to implement preventive measures, prolonged physical contact with residents is unavoidable. Therefore, their risk of infection is high [6]. These factors highlight the importance of determining the competency of long-term care facility nurses in EID prevention. In this study, competency in EID prevention was determined by considering three dimensions, namely knowledge, attitude, and skill [12,13]. In particular, competency in EID prevention involves participants’ awareness of EIDs, attitude toward implementing EID preventive measures, and perceived skill level for implementing the preventive measures.

With rapid changes in the global environment and the accompanying crisis of EIDs, competency in EID prevention must evolve to reduce the damage caused by the rapid transmission of EIDs. This study investigated the competency of long-term care facility nurses in EID prevention. The aims of this study were as follows: (1) to develop a survey for examining the competency of long-term care facility nurses in EID prevention; (2) to describe the EID prevention competency of nurses in long-term care facilities; (3) to explore the degree of knowledge, attitudes, and skills of long-term care facility nurses regarding EID prevention; and (4) to identify critical factors affecting the EID prevention competency of long-term care facility nurses. The results of this study can help long-term care facilities in developing their in-service education and training courses for infectious disease prevention as well as assist health authorities in formulating relevant preventive measures.

## 2. Materials and Methods

### 2.1. Research Design

This study used a cross-sectional design with a self-developed questionnaire. Nurses from long-term care facilities in Tainan, Taiwan, were invited to participate in the study from May to July 2021, and data were collected through online questionnaires.

### 2.2. Participants

In this study, the workplace was adopted as the sampling unit. Registered nurses who could read and write Chinese, had hands-on long-term-care practice experience, and had an active nursing license in Tainan during the data collection period were included. Nurses who could not use the Internet to complete the questionnaire were excluded. Participants were selected using a cluster random sampling method. The list of facilities was archived in Excel software, and computer-generated random codes were used to ensure the representativeness of the sample.

### 2.3. Research Instruments

The self-developed “Competency of Nurses in Long-term Care Facilities in Emerging Infectious Diseases Prevention Scale, Chinese Version” was used as the research instrument in this study. This instrument includes three scales on knowledge, attitude, and skills. In the pilot study, the questionnaire responses of 61 nurses were evaluated. After deducting an invalid questionnaire (incomplete responses), we examined 60 valid questionnaires, of which 30 were from nurses employed in residential care homes and 30 from nurses employed in nursing homes. The instrument was reviewed by 10 experts from clinical practice, government, and academia in the fields of long-term care, infectious disease prevention, and instrument development; the content validity index (CVI) of the instrument was determined. The results revealed that the overall CVI for the personal attribute and professional background domains and the knowledge, attitude, and skills subscales was 0.98, 0.97, 0.96, and 0.97, respectively. The results of the reliability test indicated that the Cronbach’s α value of the skills subscale was 0.94, indicating high internal consistency and above-average reliability. The Cronbach’s α value of the attitude subscale was 0.79, indicating excellent internal consistency and satisfactory reliability. Finally, the overall Kuder–Richardson 20 (KR-20) of the knowledge subscale was 0.63, indicating acceptable reliability.

The 59 items included in the final test were structured as follows: 23 items in the skills subscale, 12 each in the attitude and knowledge subscales, and 12 in the personal attribute and professional background domains. Questions on knowledge regarding EID preventive measures, such as transmission-related precautions, were assigned a score of 1 for a correct response and 0 for an incorrect response. Responses for questions on attitudes toward EID prevention (e.g., willingness to work during an EID epidemic) were rated on a 4-point Likert scale, with 4, 3, 2, and 1 indicating strongly agree, agree, disagree, and strongly disagree, respectively; reverse questions were scored inversely. Responses for questions on EID prevention were rated on a 4-point Likert scale based on a participant’s ability to perform a specific task (e.g., using appropriate personal protective equipment), with the scores of 4, 3, 2, and 1 indicating completely competent, mostly competent, mostly incompetent, and completely incompetent, respectively.

### 2.4. Data Collection Method and Process

The required sample size was estimated using G-Power version 3.1.9.3 software (Heinrich-Heine-Universität Düsseldorf, Düsseldorf, Germany) [14]. Given the effective response rate of the questionnaire in the pilot study and the intention to use the results of this study in an exploratory factor analysis to identify the underlying structure of numerous variables to provide information for questionnaire formulation and item simplification [15], we determined that a minimum of 230 participants was required for the study. Participants were recruited from residential care homes and nursing homes. After obtaining the relevant approval, we distributed questionnaire quick response code to nurses who agreed to participate in the survey. Data collection was performed until the required sample size was reached.

### 2.5. Research Ethics

A noninvasive survey was used in this study. To protect the rights and interests of the participants, we obtained approval from the Institutional Review Board of National Cheng Kung University Hospital for the study protocol and informed consent all the participants who participated in the online survey; anonymity was guaranteed to the participants.

### 2.6. Data Processing and Analysis

The demographics of the long-term care facility nurses and the status quo of their competency in EID prevention are presented as descriptive statistics. Analysis of variance, Student’s *t* test, and Pearson’s correlation test were used to analyze the correlation between the personal attributes of the long-term care facility nurses and their performance in the three dimensions of knowledge, attitudes, and skills in terms of EID prevention. In addition, Pearson’s correlation and path analysis were used to investigate the pairwise correlation among the three dimensions. Finally, multiple regression analysis was performed to identify the crucial predictors of the competency of the long-term care facility nurses in EID prevention.

## 3. Results

### 3.1. Demographics of Long-Term Care Facility Nurses

A total of 235 long-term care facility nurses provided valid responses (response rate = 97.1%) that were included in the analysis. Of these respondents, 104 (44.3%) worked in residential care homes and 131 (55.7%) worked in nursing homes, thus representing the proportion of the nursing workforce in long-term care facilities. The demographic factors of the long-term care facility nurses are listed in Table 1.

The results revealed that only 23.4% of the nurses in long-term care facilities had experienced an infectious disease outbreak and 39.6% of the nurses in long-term care facilities had received in-service education on infectious diseases. The majority (84.7%) of the participants were not responsible for infectious disease prevention in their facilities. The mean scores for supervisors’ understanding and approval regarding infectious disease prevention in the facilities, as perceived by the nurses, was 3.1 (SD = 0.5) and 3.3 (SD = 0.5 4) out of 4, respectively. This finding indicated that the nurses perceived their supervisors to be moderately to highly competent in infectious disease prevention. The majority (77.4%) of the nurses felt that their facility was adequate in EID preparedness.

### 3.2. Competency of Long-Term Care Facility Nurses in EID Prevention

The average total score of the nurses in long-term care facilities for the knowledge of EID prevention subscale was 8.1 (out of 12; SD = 1.8). The average rate of providing correct responses to each question (correctness rate) was only 70%, and the score index (SI) was 68. Next, the six attributes of the knowledge subscale were analyzed. Although the average hit rate of each attribute ranged from 50% to 85%, the attributes “protective measures” and “relevant laws and policies” had the highest scores. By contrast, the attributes “basic knowledge regarding the disease” and “disease symptoms” had the lowest scores. The mean score of the “attitude toward EID prevention” subscale was 37.5 (with individual scores ranging from 26 to 48). The equivalent SI was 78, which was above average. Among the three attributes of the “attitudes toward EID prevention” subscale, the nurses’ views on adherence to pandemic prevention policies and measures were the most positive (mean = 3.32), followed by those on willingness to participate in EID prevention (mean = 3.23) and awareness regarding EIDs (mean = 2.92). Finally, the mean score of the EID prevention skills subscale was 83.3 (with individual scores ranging from 59 to 92), the equivalent SI was 91. Table 2 summarizes the total scores of all the subscales.

### 3.3. Correlation between Demographics and Competency of Long-Term Care Facility Nurses

The type of long-term care facility nurses worked in was associated with large differences in the three dimensions of EID competence (knowledge: *t* = 2.3, *p* = 0.22; attitude: *t* = 1.7, *p* = 0.048; skills: *t* = 2.3, *p* = 0.022). The results indicated that the nurses from residential care homes consistently scored higher than those from nursing homes on the knowledge, attitude, and skills dimensions of EID prevention. In addition, the facility type was the only factor affecting knowledge of EID prevention.

Significant variables related to attitudes toward EID prevention included whether the participants received supervision for infectious disease prevention (*t* = 3.8, *p* = 0.006), participants’ perceived supervisors’ understanding (r = 0.3, *p* < 0.001) and approval (r = 0.4, *p* < 0.001), and adequacy of infectious disease prevention equipment (*t* = 1.7, *p* = 0.001). The nurses who were involved in infectious disease prevention and those who believed that their facility equipment was adequate generally exhibited more favorable attitudes toward EID prevention. In addition, the belief that their supervisors understood the content of infectious disease prevention or approved the prevention activities they implemented significantly promoted the nurses’ positive attitudes toward EID prevention.

Finally, the participant’s age (r = 0.2, *p* = 0.029), marital status (F = 11.8, *p* < 0.001), years of nursing work experience (r = 0.2, *p* = 0.001), years of working in long-term care facilities (r = 0.2, *p* = 0.001), being in charge of infectious disease prevention (*t* = 2.7, *p* = 0.002), and perceived supervisor’s understanding (r = 0.3, *p* < 0.001) and approval (r = 0.3, *p* < 0.001) were correlated with the skill dimension of EID prevention. Among these variables, age, years of nursing work experience, years of working in long-term care facilities, and perceived supervisors’ understanding and approval were positively correlated with the participants’ EID prevention skills. In addition, the nurses who believed that their facility’s infection prevention equipment was adequate were more likely to implement EID prevention measures. Furthermore, the analysis of the participants’ marital status performed using the Scheffé test indicated that the married and divorced nurses were more adept at EID prevention compared with their unmarried counterparts. The overall results of the analysis are presented in Table 3.

### 3.4. Pairwise Correlation of the Competency Dimensions of Long-Term Care FacilityNurses in EID Prevention

Significant correlations were observed among the different dimensions of the long-term care facility nurses’ competence in EID prevention except for knowledge and skills (r = 0.038, *p* = 0.557). A positive correlation was noted between knowledge and attitude (r = 0.174, *p* = 0.008) and between attitude and skills (r = 0.32, *p* < 0.001).

**Figure 1:**
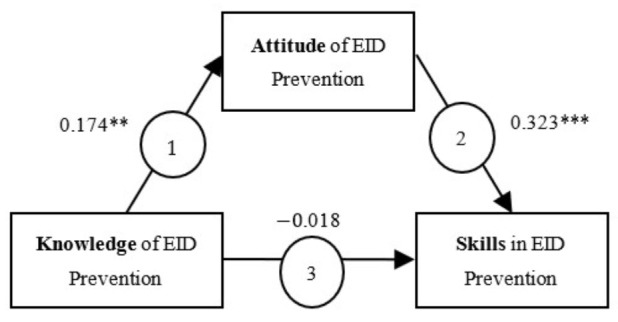
Path analysis diagram of the three dimensions of competency in emerging infectious disease (EID) prevention. ** *p* < 0.01; *** *p* < 0.001.

On the basis of the results of correlation analysis, we performed path analysis to examine the causal relationship among the three dimensions of the nurses’ competence in EID prevention and to confirm whether the attitude dimension exerted a mediating effect and whether knowledge regarding a mediator indirectly affected the skill related to a mediator. The knowledge dimension significantly affected the attitude dimension (Table 4). The effect of the attitude dimension on the skill dimension was significant; however, the effect of the knowledge dimension on the skill dimension was nonsignificant. Hence, as depicted in Figure 1, two of the three paths had a significant coefficient, indicating that attitudes must be first improved for knowledge to exert an effect on skills.

### 3.5. Significant Predictors of the Competency of Long-Term Care Facility Nurses in EID Prevention

To examine whether an independent variable (i.e., the personal attributes and professional background of the long-term care facility nurses and the three dimensions of their competence in EID prevention) would be a significant predictor of the dependent variables (i.e., the three dimensions of the nurses’ competence in EID prevention), we performed a stepwise regression analysis. The results are summarized in Table 5. We observed that the attitude toward EID prevention was a significant predictor of the knowledge dimension because the attitude toward EID prevention explained 3% of the variance in the knowledge dimension (β = 0.174, *p* < 0.01). Similarly, significant predictors for the attitude dimension were perceived supervisors’ approval (β = 0.277, *p* < 0.001), EID prevention skills (β = 0.193, *p* < 0.01), and being in charge of infectious disease prevention (β = 0.125, *p* < 0.05), which together explained 22.3% of the variance. Finally, significant predictors for the skills dimension were perceived supervisors’ approval (β = 0.197, *p* < 0.01), marital status (β = 0.307, *p* < 0.001), and attitudes toward EID prevention (β = 0.279, *p* < 0.001), which together explained 24.8% of the variance.

In summary, the aforementioned six variables were the primary predictors of the three main dimensions of the long-term care facility nurses’ competence in EID prevention and explained 3–24.8% of the total variance. The participants who received approval from their supervisors to implement EID prevention measures, were married or divorced, had more knowledge regarding the disease, were more positive regarding EID prevention, were more confident in implementing EID prevention measures, and had experience in implementing infectious disease prevention measures at the facility were more competent in implementing EID prevention activities.

## 4. Discussion

### 4.1. Feasibility of the Research Instrument for Determining the Competency of Long-Term Care Facility Nurses in EID Prevention

Because of the unavailability of an effective research instrument to examine long-term care facility nurses’ competency in EID prevention, we developed the instrument for this study. The Competency of Nurses in long-term care facilities in EID Prevention Scale, Chinese Version was well received by experts from clinical practice, government, and academia in terms of its accuracy and the appropriateness of its content. In addition, the comparison of reliability between the questionnaire used in the pilot study and the current questionnaire used in the formal study indicated acceptable-to-satisfactory internal consistency. The high internal consistency is probably due to the use of the stratified sampling method in this study, in which the samples were divided according to the type of institution. Such a research design reduced both sample variability and response instability, thus ensuring the credibility and stability of the research instrument when collecting data from the long-term care facility nurses.

The scale had a high CVI and internal consistency, confirming its value and potential for development. For future research, the instrument can be further developed through the inclusion of qualitative interviews, the Delphi method, confirmatory factors, and reliability testing to shorten the scale while maintaining or improving the high discriminatory power and accurate reflection of the constructs.

Because EIDs are constantly changing and their transmission paths are unpredictable, the knowledge dimension of the questionnaire should be continually updated. Situations that evolve over time and in different settings as well as knowledge derived from empirical bases imply that both the concepts and content of questions should be updated in real time.

### 4.2. Competency of Long-Term Care Facility Nurses in EID Prevention

The participants’ inconsistent self-assessment on the three dimensions did not match distributions predicted using the knowledge-attitude-behavior model widely used in the field of health education. The model developed by Baranowski et al. [16] assumes that an individual’s behavioral changes are affected by knowledge. The accumulation of knowledge by individuals changes their attitudes, thus promoting the adoption of appropriate behaviors. However, in our study, the results of path analysis indicated that knowledge alone cannot directly change skills unless the nurse has a positive attitude toward prevention and recognizes the importance of prevention skills. Therefore, nurses should not only learn regarding EIDs but also have a positive attitude while creating prevention plans. The research team recommends that nurses in long-term care facilities should make greater effort to acquire knowledge regarding preventing EIDs and that it is also important to promote their willingness through incentives. In addition, in the future, continuing education instructors should pay attention to the appropriateness of the course content [17] to increase participation rates and thus improve nurses’ knowledge in certain areas [18].

Previous research indicated that supervisors can set an example of self-confidence and self-regulation through authentic leadership, therefore promoting the development of positive work attitudes in their subordinates, and that recognition by supervisors can positively affect subordinates’ behavior [19,20]. This finding is in agreement with our observation that perceived supervisor approval was the most significant predictor of the three dimensions of the long-term care facility nurses’ competence in EID prevention, accounting for 13.1% of the variance. The more the supervisor acknowledged the nurses’ implementation of EID prevention measures, the better they were able to perform related tasks. Supervisors of long-term care facility nurses should acquire the right knowledge and skills for EID prevention to strengthen their recognition and promotion of the implementation of appropriate interventions. When long-term care facility supervisors clarified the meaning of EID prevention and promoted the understanding of staff regarding their respective roles, the nurses demonstrated higher work engagement and confidence, had more positive attitudes toward achieving work goals, and were more compliant with relevant regulations, thus effectively improving the quality of services provided by the facility. To enable nurses to realize the importance of EID prevention, they should be helped to acquire accurate and appropriate knowledge regarding EID prevention. In addition, the Regulation on Registration and Continuing Education of Medical Professionals requires nurses in Taiwan to complete 120 h of continuing education courses every 6 years. Therefore, measures to improve nurses’ attitudes should be developed and used in continuing education courses on infection control in the future. Such courses can promote a deeper understanding of EID prevention among long-term care facility nurses and help them recognize the importance of EID prevention, which in turn, would help them develop the appropriate EID prevention skills. This finding is consistent with the results of studies conducted by Bartzokas and Slade [21], Ben-Ari [22], and Berhe et al. [23] who reported that the dimensions of knowledge and skills can affect each other and that the appropriate knowledge can improve an individual’s skills.

Compared with the mean score of public health nurses in EID prevention reported by Cai [24], which was 15.89 (SI = 63.5) for the knowledge dimension, 48.24 (SI = 68.9) for the attitude dimension, and 82.84 (SI = 75.3) for the ability dimension, the scores of the long-term care facility nurses in our study were considerably higher. This result suggests that nurses working in different settings, such as long-term care facilities or health centers, have different competencies in EID prevention. A comparison of the findings of this study with those reported by Cai indicated that nurses working in long-term care facilities were better able to perform their EID prevention tasks.

### 4.3. Limitations of Self-Report Questionnaires

The main disadvantage of using self-report questionnaires is the possibility of social desirability bias because respondents may not answer truthfully but in a socially acceptable manner. In addition, response bias, which refers to the tendency of an individual to answer in a certain manner regardless of the question, may be encountered. This study was conducted in Taiwan; thus, its results cannot be generalized to long-term care facilities in other countries.

The nurses were asked to voluntarily participate in this study, and those who participated might be significantly more motivated compared with those who refused. The personal characteristics of the participants in this study were significantly more positive than those of the refusers; this might have affected the results of the attitude construct. In addition, the accuracy of the results of the skills construct should be validated because the construct still depends on the effect of the presence of the actual cases of EIDs in the facility. If participants do not have experience managing EIDs, they may fill in responses based on hypothetical thinking. Thus, the overall scores on the attitudes and skills subscales may be overestimations and not accurately reflect the attitudes and skills of long-term care facility nurses regarding EIDs. However, the survey period coincided with the severe global outbreak of a specific infectious disease, COVID-19, which required nurses to acquire extensive knowledge regarding pandemic preparedness. The results of this study may be overstated due to the respondents’ seeking to meet public expectations that healthcare workers should be trained in epidemic preparedness.

## 5. Conclusions

The Competence of Nurses in long-term care facilities in EID Prevention scale developed in this study confirmed its value and potential with a high CVI and internal consistency. For competence in EID prevention, nurses’ knowledge was insufficient, but they showed moderate to very positive attitudes and were also confident that they had the necessary skills. An examination of the dimensions’ degree in EID prevention indicated that the long-term care facility nurses who had knowledge regarding EIDs exhibited more positive attitudes toward EID prevention, properly implemented prevention measures, and developed appropriate EID management skills. In addition, perceived supervisor’s approval, marital status, attitudes toward EIDs prevention, EID prevention skills, knowledge of EIDs, and being in charge of infectious disease prevention were significant predictors of the three dimensions of EID prevention. The results can serve as a reference for the development of continuing education programs for infectious disease prevention in long-term care facilities. A problem identified in this study is the difficulty in interpreting the long-term care facility nurses’ perception regarding EID prevention. In the future, additional qualitative interviews should be conducted to understand participants’ perceptions, to improve their acceptance of national or institutional policies in EID prevention.

## Figures and Tables

**Table 1:** Demographic characteristics of long-term care facility nurses (*N* = 235).

Variable	*N*	%	Mean	Min	Max	Median
			(Standard Deviation)			
**Long-term care facilities type**						
Residential care homes	104	44.3				
Nursing homes	131	55.7				
**Age**			34.4 years (SD = 10)	20	65	33
**Marital status**						
Unmarried	122	51.9				
Married	102	43.4				
Divorced	11	4.7				
**Working years (Nursing)**			11.6 years (9.8)	0.3	42	10
**Working years (LTC)**			6.9 years (6.8)	0.3	35	4.4
**Infectious disease care experience**						
Yes	55	23.4				
No	180	76.6				
**In-service education on infectious diseases**						
Yes	93	39.6				
No	142	60.4				
**In charge of infectious disease prevention**						
Yes	36	15.3				
No	199	84.7				
**Supervisor’s perceived understanding ^a^**			3.1 (0.5)	1	4	3
**Supervisor’s perceived approval ^a^**			3.3 (0000.5)	2	4	3
**Infectious disease prevention equipment**						
Adequate	182	77.4				
Inadequate	53	22.6				

^a^ “Supervisor’s perceived understanding” and “Supervisor’s perceived approval” were rated on a 4-point Likert scale, with scores of 4, 3, 2, and 1 indicating high understanding and agreement, moderate understanding and agreement, no understanding, and no understanding and no agreement, respectively. EID, emerging infectious diseases.

**Table 2:** Analysis of questionnaire scores on the competency in EID prevention among long-term care facility nurses (*N* = 235).

Scale/Subscale	Items	Correctness Rate	Mean (Standard Deviation)	Average Score ^d^	Order
		%			
**Knowledge scale of EID prevention ^a^**	**12**		8.1 (1.8)		
Basic knowledge regarding the disease	2	50.0	1.0 (0.8)	0.5	5
Pathogenic factors and transmission routes	2	63.6	1.3 (0.7)	0.65	4
Disease symptoms	2	47.7	1.0 (0.7)	0.5	6
Treatment	2	70.6	1.4 (0.6)	0.7	3
Protective measures	2	86.4	1.7 (0.5)	0.85	1
Relevant laws and policies	2	82.6	1.7 (0.5)	0.85	2
**Attitudes scale toward EID prevention ^b^**	**12**		37.5 (4.9)	3.1	
Nurses’ perception of EIDs	5		14.6 (2.6)	2.92	3
Willingness to work during EID epidemic	3		9.7 (1.5)	3.23	2
Feelings about compliance with epidemic prevention policies and preventive measures	4		13.3 (1.7)	3.32	1
**EID prevention skills scale ^c^**	**23**		83.3 (7.8)	3.6	

^a^ For questions on knowledge regarding EID prevention, a correct answer was assigned a score of 1 and an incorrect answer was assigned a score of 0. The higher the score was, the more accurate was the knowledge of the long-term care facility nurse regarding EID prevention, ^b^ The attitudes toward EID Prevention were scored on a 4-point Likert scale according to the participant’s degree of agreement with the statement, with scores of 4, 3, 2, and 1 representing strongly agree, agree, disagree, and strongly disagree, respectively. Reverse questions were scored in a reverse order. The higher the score was, the more positive was the attitude of the long-term care facility nurse toward EID prevention, ^c^ The skills in EID prevention were scored on a 4-point Likert scale according to the participant’s ability to complete a certain task, with scores of 4, 3, 2, and 1 representing totally competent, mostly competent, mostly incompetent, and totally incompetent, respectively. The higher the score was, the more skilled the long-term care facility nurse was at implementing EID prevention measures, ^d^ Average score = Average number/number of questions. EID, emerging infectious diseases.

**Table 3:** Correlations among demographic attributes and EID prevention in long-term care facility nurses (*N* = 235).

Variable	*N*	Knowledge			Attitudes			Skills		
		Mean (Standard Deviation)	Test Statistic ^a^	*p*-Value	Mean (Standard Deviation)	Test Statistic ^a^	*p*-Value	Mean (Standard Deviation)	Test Statistic ^a^	*p*-Value
**Long-term care facilities** **type**	235		*t* = 2.3	0.022		*t* = 1.7	0.048		*t* = 2.3	0.022
Residential care homes	104	8.2 (1.6)			37.8 (5.3)			84.9 (7.2)		
Nursing homes	131	8.0 (2.0)			37.5 (4.6)			82.2 (8.0)		
**Age**	235		r = 0.0	0.926		r = −0.1	0.199		r = 0.2	0.029
**Educational background**			F = 1.2	0.324		F = 1.0	0.418		F = 1.1	0.361
Senior (High School)	10	7.7 (2.1)			36.7 (6.4)			82.9 (9.2)		
Associate degree	83	8.0 (1.7)			37.0 (5.2)			84.1 (7.5)		
Bachelor’s degree	133	8.0 (1.9)			38.0 (5.0)			83.2 (7.1)		
Master’s degree	9	9.1 (1.4)			39.1 (5.3)			79.5 (15.4)		
**Marital status**			F = 1.6	0.206		F = 1.7	0.178		F = 11.8	<0.001
Unmarried	122	7.9 (1.9)			38.2 (4.6)			81.3 (7.2)	Scheffe test 2,3 > 1	
Married	102	8.2 (1.7)			36.9 (4.9)			85.1 (7.9)		
Divorced	11	8.6 (2.1)			37.6 (7.2)			90.1 (4.4)		
**Job title**			F = 0.881	0.416		F = 2.94	0.054		F = 1.31	0.054
Manager	34	7.7 (2.0)			39.3 (4.3)			85.1 (9.2)		
Practice Nurses	193	8.1 (1.8)			37.2 (5.0)			83.0 (7.6)		
Other	8	7.9 (1.1)			39.1 (2.2)			85.1 (5.3)		
**Working years (Nursing)**			r = −0.0	0.904		r = −0.1	0.167		r = 0.2	0.001
**Working years (LTC)**			r = −0.1	0.080		r = −0.1	0.504		r = 0.2	0.001
**Infectious disease care experience**			*t* = −1.2	0.995		*t* = 0.2	0.074		*t* = −0.2	0.486
Yes	55	7.9 (1.9)			37.7 (5.4)			83.2 (7.6)		
No	180	8.1 (1.8)			37.6 (4.8)			83.4 (7.8)		
**In-service education on infectious diseases**			*t* = −1.2	0.058		*t* = 0.8	0.866		*t* = 2.1	0.066
Yes	93	7.9 (1.6)			37.9 (4.8)			84.7 (7.2)		
No	142	8.2 (2.0)			37.4 (5.0)			82.5 (8.1)		
**In charge of infectious disease prevention**			*t* = −1.6	0.977		*t* = 3.8	0.006		*t* = 2.7	0.002
Yes	36	7.6 (1.8)			39.8 (3.6)			85.9 (5.6)		
No	199	8.1 (1.8)			37.2 (5.0)			83.0 (8.0)		
**Supervisor’s perceived understanding**			r = 0.0	0.702		r = 0.3	<0.001		r = 0.3	<0.001
**Supervisor’s perceived approval**			r = −0.1	0.416		r = 0.4	<0.001		r = 0.3	<0.001
**Infectious disease prevention equipment**			*t* = 0.6	0.392		*t* = 1.7	0.001		*t* = 3.1	0.237
Adequate	182	8.1 (1.9)			37.8 (5.2)			84.2 (7.9)		
Inadequate	53	7.9 (1.6)			36.8 (3.3)			80.6 (6.8)		

^a^ Test statistics: r refers to the Pearson’s correlation coefficient, *t* refers to the *t* test, and F refers to one-way analysis of variance. EID, emerging infectious diseases.

**Table 4:** Path analysis for EID prevention knowledge, attitudes, and skills among nurses in long-term care facilities (*N* = 235).

Dimension	R	R^2^	Standardized Regression Coefficients β	*t*	*p*-Value
“Knowledge dimension” vs. “Attitude dimension”	0.174	0.030	0.174	2.694	0.008
“Knowledge dimension” vs. “Skill dimension”	0.320	0.102	−0.018	0.279	0.780
“Attitude dimension” vs. “Skill dimension”	0.320	0.102	0.323	5.110	<0.001

EID, emerging infectious diseases.

**Table 5:** Critical factors affecting the EID prevention competency of nurses in long-term care facilities (*N* = 235).

Variables	Knowledge	Attitudes	Skills
	Standardized Regression Coefficients β	Standardized Regression Coefficients β	Standardized Regression Coefficients β
Supervisor’s perceived approval	N/A	0.277 ***	0.197 **
Marital status	N/A	N/A	0.307 ***
Attitudes toward EIDs prevention	0.174 **	N/A	0.279 ***
EID prevention skills	N/A	0.202 **	N/A
Knowledge of EIDs	N/A	0.193 **	N/A
In charge of infectious disease prevention	N/A	0.125 *	−0.008
long-term care facilities type	−0.042	0.022	−0.096
Age	N/A	N/A	−0.120
Working years (Nursing)	N/A	N/A	0.012
Working years (LTC)	N/A	N/A	0.031
Supervisor’s perceived understanding	N/A	0.029	0.105
Infectious disease prevention equipment in facilities	N/A	0.017	N/A
R^2^	0.030	0.223	0.248
Adjusted R^2^	0.026	0.210	0.238
*t*	2.694 **	2.083 *	4.500 ***

N/A not applicable; * *p* < 0.05; ** *p* < 0.01; *** *p* < 0.001. EID, emerging infectious diseases.

## Data Availability

Because the development of the instrument used in this study is ongoing and future contributions are expected, the data section is currently unavailable.
